# Novel intelligent architecture and approximate solution for future networks

**DOI:** 10.1371/journal.pone.0278183

**Published:** 2023-03-01

**Authors:** Akram Sarhan, Mahdi Jemmali

**Affiliations:** 1 Department of Information Technology, College of Computing and Information Technology at Khulis, University of Jeddah, Jeddah, Saudi Arabia; 2 Department of Computer Science and Information, College of Science at Zulfi, Majmaah University, AL-Majmaah, Saudi Arabia; 3 MARS Laboratory, University of Sousse, Sousse, Tunisia; 4 Department of Computer Science, Higher Institute of Computer Science and Mathematics, University of Monastir, Monastir, Tunisia; Cleveland State University, UNITED STATES

## Abstract

Private networks have become popular for secure data sharing and anonymous communication in many domains: enterprise environments, military, journalism, telecommunication, healthcare, to name a few. It has been used with or without internet connection. Its primary purpose is to provide confidentiality, bypass unlawful activities, and protect against common threats such as interception, modification, and censorship. In addition, several private network technologies exist to support secure communications. However, they mostly rely on encryption only. The transmitted data is classified into different confidentiality levels. This research presents a smart private network architecture scheme that transmits constraint-based classified packets. The main directive of this work is the proposed constraint. This constraint is meant to enforce that if two packets belong to the same confidentiality level, they can’t be transmitted through the two routers simultaneously. Therefore, the studied problem is an NP-hard problem. This paper presents the following contributions: (i) proposes a new architecture paradigm for outsourcing a constraint-based multi-classified data sharing securely and transmitted through two routers; (ii) introduces several algorithms to prove the feasibility for this NP-Hard problem; and (iii) implements the algorithms solutions using C++ and compares their performance. Different metrics are used to measure the performance of the proposed algorithms. Randomized Longest Transmission time first algorithm $\bar{RLT}$ scored the best algorithm with a percentage of 73.5% and an average gap of 0.002 according to the experimental results. It is remarkable worthy to note that the execution time of all the algorithms is less than 0.001 s.

## 1 Introduction

The “communication gap” between the experts who are working on network development and those working on technology development for security purposes has led to a significant lack of both methodologies to manage the complexity of security needs and the flexibility of security methods deployment. Network security design relies on the Open System Interface (OSI) model, which provides protocol standardization, flexibility, and modularity [1] In fact, the Internet revolution which has been developed in the past 20 years, classified computer network development and advancement into several stages [2]. The so-called “Active Networks” programmable functionality started in the 1st stage. In the second stage, open interfaces were developed to communicate between the control and data planes. Finally, Network Virtualization (NV) for better routing flow and the “OpenFlow API and network operating systems (N.O.S.s)” was developed in other stages.

The exponential increase in data production resulted in a need for emerging solutions for data management using artificial intelligence (AI), machine learning (ML), and analytical tools. However, despite the importance of the produced information and their analysis to deal with issues concerning pandemic risks, disease prevention, or climate changes, exposing these data through any stage during their lifecycle becomes a serious cybersecurity concern because of the expandability of the Internet.

Based on TCP/IP protocol suite, IPv4 and IPv6 addressing, the Internet has expanded into a worldwide network (WWW), which has become universally accessed and enabled billions of devices and people to connect, exchange, and share information [3]. However, the anonymous nature of the Internet, including its architectural design, failed to provide a suitable solution to address many security, privacy, and performance issues, leading to a significant increase in cyberattack incidents [3, 4]. Technology virtualization includes Software-Defined Networking (SDN) and Network Function Virtualization (NFV) are considered new approaches for building, designing, and managing future programmable networks to overcome the density of the internet infrastructure.

Recently, a new direction or movement of the Internet has taken place owed to the progress in the wireless network domain and innovative mobile device capabilities. Furthermore, infrastructure less wireless network technology like mobile ad hoc network (MANET) is a decentralized network that does not rely on routers or access points to forward the data; instead, it is being formed from mobile nodes that can be created and joined “on the fly” and forward the data based on network connectivity, and the routing algorithms [5]. Despite the various applications that MANET support, it poses several challenges and is vulnerable to various attacks [6].

On a more advanced level, the commercialization of the Internet and the introduction of new technologies like the fifth generation (5G), transformed the communication architecture or the cyber-physical to provide connectivity to connect people, things, devices, applications, transport, and even cities. However, anything that relies on emerging concepts and technologies like everything IP, everything Data, Long Term Evolution (LTE), and IoT, have brought challenges concerning security, privacy, and trust [7]. For example, nearly 90 percent of the infrastructure has been owned by the private sectors, which brings a concern to safeguard data outsourced or routed by U.S.A military or government organizations. To answer the demand of future computer networks, several experimental networking projects were designed for next-generation networks (NGNs), For instance, the global environment for network innovations (GENI), the PlanetLab, and the project of future Internet design (FIND) program. However, the complexity and weakness of the current network protocols make it difficult to keep up with the rapid growth and advancement in communications and applications. For instance, the system administrators and users have limited knowledge of the networking infrastructure. This, in turn, resulted in the inability to apply security tools to protect the environment due to the complexity and weakness of the current internet network protocols.

Literature has well studied the network packets timing, scheduling, and movements problems. Network Packet scheduler specifies the packets that need to transit and those that need to wait at each time. Several sequential and parallel scheduling algorithm solutions have been proposed to minimize packet routing timing and maximize the queue size [8]. The advancement in innovative communications technologies in various domains has presented a vast range of applications in several areas, such as environmental surveillance, biomedicine, and video security monitoring. Thus additional issues to the network routing problems like latency, energy consumption, and security have emerged. Several routing scheduling algorithms methods, policies, and heuristics have been proposed to deal with issues related to routing security and privacy, optimization, delay, energy consumption, recovery time, and overhead in many modern network technology such as WirelessHART Mesh Networks, Wireless Sensor Networks, Industrial Wireless Mesh Networks, and Wireless Body Sensor Networks [9].

Protecting networks depends on ensuring the following security requirements: confidentiality to protect data from unauthorized users, integrity to protect data from unauthorized modification, and availability to protect data from being damaged or lost. Furthermore, the damage of the communication infrastructures can cause data to be damaged during war or natural disasters such as earthquakes, tsunamis, and floods. Therefore, there is a need for data control solutions in such emergencies [10]. The ongoing evolution of artificial intelligence (AI) technologies makes it possible to create emergency data allocation algorithmic techniques that deal with such incidents as proposed in [11] using the scheduler and router-based window constraints. The window is used by an intelligencer via a scheduler to control the network and create a “static urgent window pass” to reserve a time slot or pause accessing the router when dealing with scheduling problems during data transmission of confidential and urgent data based on given constraints [10, 12]. Failure to provide any security requirements leads the Networks architecture to be prone to numerous cybersecurity attacks such as eavesdropping, sybil, selective, smurf, sinkhole, masquerading, byzantine, location disclosure attacks, blackhole, wormhole, and man-in-the-middle [13].

Access control contributes to the security requirements of securing the network in terms of confidentiality, integrity, and availability (CIA). However, such security requirements pose some limitations in dynamic environments. Therefore, a new generation of access control mechanisms has been demeaned to cope with today’s dynamic digital environments. The classification of information into top-secret, secret, confidential, and unclassified has existed for a long time. Several Multilevel security policy models and systems have been proposed. Several problems and concerns have been pointed out concerning the practicality of MLS. They are summarized as follows. (i) The cost and difficulty of building them; (ii) the need to rebuild several applications to cope with MLS; and (iii)the complexity of the classification of data. With the technological improvements, several variations of traditional and untraditional access control mechanisms have been proposed to safeguard against unauthorized access. These mechanisms are encrypted-based or encryption-independent. Discretionary Access Control (DAC), Mandatory Access Control (MAC), Role-Based Access Control (RBAC), Attribute-Based Access Control (ABAC), and Context-Aware role-based Access Control (CAAC) are all access control techniques and frameworks [14].

The main motive of this research is to advance state-of-the-art research in computer networks by proposing a future network vision that can be highly secured. A major problem is stopping or minimizing data leakages for sensitive outsourced information, which has become a significant problem nowadays. It also proposes a model that is capable to protect data of individuals in particular fields, for instance, during critical coverages for individuals (e.g., Journalists, Media outlets). Furthermore, the model investigates nontraditional methods or techniques existed in state-of-the-art. For example, our method was a reduction of an NP-Hard problem that deals with two machines. This paper aims to propose an intelligent private network architecture that disseminates classified network packets based on the constraint. It also proposes a robust network paradigm that relies on two routers to address critical data privacy issues in particular environments and circumstances. The proposed scheme outsources data securely and privately in a critical environment. The approach has the following advantages: (i) It introduces an idea or vision for a secure future network using intelligent algorithms; (ii) Supports individuals (e.g, Journalists, Media outlets) to securely and privately exchange sensitive information with their hosts; (iii) provides a unique two-router network architecture for transmitting network packets based on a constraint-based classification; (iv) Presents algorithms using known and unknown techniques such as dispatching rules, local insertion search, randomization method, and lifting procedure; (v) Introduces a group of heuristic algorithms that deal with an NP-hard problem and applies it in the computer network area; (vi) Solves the problem in an acceptable optimal time, as it is shown in the result section. The disadvantages of our approach are listed as follows: (i) The two-router problem in our scheme is a reduction of a known NP-hard problem. Thus, solving such a problem using multiple hops could be time-consuming and might require more effort and techniques to heuristically solve it using algorithms with *O*(*n*^3^). (ii) The time complexity to solve the problem relying on multiple hops could be very complicated because using two routers in our scheme problem reduces a known NP-hard problem. (iii) The exact solution for the problem requires a lower bound to be used in a branch-and-bound algorithm in the future; thus, an optimal solution can be given for our problem using the developed algorithm in this paper. Overall, this paper aims to propose a smart private network architecture that disseminates classified network packets based on the constraint.

It includes eight sections presented as follows. First, we introduce the related work in section 2. Then, the studied problem is described in Section3. Third, we present the novel proposed architecture and the different components in Section 4. Next, a detailed description of a novel lifting procedure is presented in Section 5. Fifth, we present the proposed solution algorithms in Section 6. Furthermore, we show the experimental setup and performance comparison between the algorithms in Section 7. Finally, some concluding remarks with reference to the future work are included in Section 8.

## 2 Literature review

Literature has investigated threats for the transmitted data across all network layers in various distributed computing systems, applications, and technologies. The analysis of the threats showed attacks against one or more of the C.I.A. triad- confidentiality, integrity, availability, including authentications, non-repudiation, and in various emerging and intelligent communications and technologies domains. For example, attacks on wireless mobile ad hoc networks are investigated in [15], attacks on wireless sensor networks [16], and cloud computing environments are discussed in [17]. Furthermore, due to a lack of end-to-end novel security solutions, several attacks have been against evolving technologies, for instance, those depending on artificial intelligence (AI) [18], augmented reality (AR) [19], Software-Defined Networking (SDN) paradigm [20] and Blockchain addressed in [21]. Attacks against new business models and e-health applications presented in [22].

The internet protocols have been viewed as a set of layers or protocol stack described according to the open system’s interconnections (OSI) into a seven-layered network [23]. Several protocols such as IPSec, SSL, and DNSSEC which have been proposed to provide end-to-end security solutions. For example, since routing information is subject to being intercepted or modified when routed through unknown networks, IPSec protocol has been developed to ensure end-to-end encryption for the Internet protocol (IP) user data and traffic.

The geographical distribution of various enterprises, sites and offices worldwide has led to the need for point-to-point or site-to-site private networks and secure connections. Virtual private network (VPN) has become popular for creating a private network to share resources and disseminate data over insecure networks. The secure channels in the VPN use the IPSec protocol. Unlike VPN, Virtual Private LAN services (VPLS) have been designed to control new evolving patterns, such as NFV and SDN. It has been popular for providing multipoint-to-multipoint connectivity, including other features, such as robust security and low operational cost. Still security in VPLS is a big concern [24].

Mobile ad hoc network MANET has been adopted due to its numerous advantages. The routing functionality in MANET is integrated into its nodes. Both topology-based and position-based routing protocols have been adopted in MANET. However, due to its decentralized nature and lack of efficient algorithm, designing routing protocol in MANET has been considered a complex issue [25]. In [26], the authors extended the greedy perimeter stateless routing protocol (GPSR)to enhance the position-based routing protocols for MANET. In addition, they used fuzzy logic to adjust the lifetime of entries in the nod’s neighborhood matrix (IFPE).

The invention of 5G mobile communications and the advancement in computer network architectures, including SDN, NFV, and smart communication wireless devices, has made traditional data management systems unable to handle massive data. Accordingly there is a need to process higher data rates, guarantee lower data latency, and manage big data. To these ends, newer network architecture design and better algorithms embedded with security and decision-making capabilities are required [27]. Furthermore, Network routing should deal with policy compliance and avoid the dependence on algorithmic optimization [3]. For example, they used a scheduler in their scheme [12] to prioritize highly confidential classified packets in circumstances where the guarantee of the transmission of such packets could be uncertain. Furthermore, the authors used a single router with a static window pass [10, 12] to experiment with several proposed heuristics and thus prove the practicality of their work. In addition, in [28], the authors used the scheduler to solve the problem of identical routers into network.

Data are publicly classified into several classes: top-secret, secret, restricted, and public, and others use such other terms like regulatory, public, confidential (highly confidential), and internal. Data classification using security policies has been used in many domains military, business, and healthcare [29]. For instance, a secure data and identity multilevel security outsourcing scheme are proposed in [30].

Multilevel security (MLS) controls the disclosure of data in trusted and unsafe environments. Only authorized individuals can access, modify, or delete data. The current network behavior is static, which makes the network sluggish in unstable network environments (e.g., traffic patterns or topology changes, or link failures)-thus there is a need for a Multilevel security policy that can be adopted under any circumstances [31]. Ali et al. proposed a blockchain-based IoT network multilevel security architecture that provides multilevel protection of data. The scheme uses cipher ChaCha20 and cellular automata to gain more security and randomness. The same authors claimed that their scheme enhances security and protects against all kinds of attacks by providing multiple levels of encryption; however, their scheme is not flexible and cannot minimize the chances of leakages [32]. In [33], the authors proposed a scheme that transmitted the data securely between cloud service and Internet of vehicles (IoV) devices. Their scheme uses an M-tree-based elliptic curve and digital signature algorithm (ECDSA) to provide key management for multilevel security infrastructure. In [34], The authors proposed a MLS scheme that enforced the flow policy of information among the inter-node within the network to minimize chances for attacks. Their scheme enforces the MLS policy on the software-defined networks SDN switches by moving the job to the controller. Unlike the proposed MLS methods, our scheme MLS policy ensures the absence of transmitting an identical level of secure information simultaneously.

Several schemes used reputation systems mechanisms to ensure a trusted routing environment. In [35], the authors proposed a trusted-based routing protocol model that recommends the trusted routing node to improve security. In [36, 37] authors embed a trust-based mechanism in the routing path for routing path scheduling. Other authors used Blockchain and trusted public key protocols [38] to create decentralized inter-domain trusted routing systems and use smart contracts [39] to follow a trusted route to the destination. Detection of malicious nodes using reinforcement learning by automatically discovering the packets number transmitted to node’s neighbor nodes was studied in [40]. In [41], the authors proposed a scheme to ensure the privacy of the source location to maintain safety time. The scheme selects multiple phantom nodes based on a dynamic routing generation process, adds a randomly directed path, and transmits the packets through different phantom nodes to ensure security [42].

Even though, there is a strong need for a multilevel secure data dissemination solution in a military-based environment, not enough research and investment in this domain exist-despite the need to use such a solution in the current era where collaboration between businesses and governmental organizations becomes necessary. Furthermore, even though several access-control mechanisms have been deployed for secure data dissemination [30, 43–45], they are not fully practical in dealing with the multilevel protection of classified big data or data streams needed in a military-based environment. In fact, the current paper proposes a model that relies on two router-based architecture to schedule securely and then disseminate conflict-based multilevel packet security in a critical situation. In addition, other researches related to the representation of the network traffic are developed in [46, 47].

The suggested techniques presented in this paper can be exploited and operated to be adopted to the problems developed in [48–52]. We believe that our approach is important since it provides optimal security due to its multilevel security that minimizes the level of leaked information in case of cybersecurity attacks compared to other approaches. For example, the highly secure dissemination of packets in our approach justifies our important architectural choice of two routers since two packets that belong to the same level of security are prohibited from being transmitted at the same time, and this only can be accomplished in the main time through more than one router. Thus, if an adversary could capture one highly secure packet at one point through one router, it would not be able to capture a second one simultaneously since our architecture promotes transmitting classified packets with multiple levels of security. Additionally, a review of the literature showed the lack of research on this NP-Hard problem, and we were the first to present it in [53]. Finally, we introduced several heuristics that can help reach metaheuristics or an exact solution for the problem in the future. The proposed algorithms in [54–60].

Unlike many researches that focused on solving the problem of exchanging or outsourcing data securely and privately at the application layer and try to solve it using known techniques or traditional methods to protect outsourced information, our innovative approach addresses the problem from a different point of view and study the problem at the network layer and propose heuristics solution reduced from known NP-Hard problem and apply in the network security field. Furthermore, the presented algorithms in [53] were based only on the dispatching rules; however, this paper uses several techniques for the problem, such as dispatching rules, the local insertion search, the randomization method, and the lifting procedure.

## 3 Problem description

The problem in focus is described as follows. In a private network wherein several files must be transmitted through two routers, it is assumed that each group of files is classified in a confidentiality level denoted by *Cl*_*i*_ with *i* = {1, ⋯, *n*_*Cl*_} where *n*_*Cl*_ is the number of confidentiality level. These files will be divided into packets, and each packet related to each file will be classified into the corresponding *Cl*. The total packets are grouped in the set *PT*. Thus, we denote *n*_*pt*_ for the number of packets.

The index of the packet is denoted by *j* and the packet is denoted by *Pt*_*j*_. The confidentiality level of the packet *Pt*_*j*_ is denoted by *Clp*_*j*_. The packet *Pt*_*j*_ has an estimated transmission time (Time of Packet Transmission) denoted by *tp*_*j*_. After scheduling the packet *j*, the cumulative transmission time on the first router *Ro*_1_ is denoted by $Tc_{j}^{1}$, and on the second router *Ro*_2_ is denoted by $Tc_{j}^{2}$. This cumulative transmission time represents the finishing transmission time of packet *j*.

*PT*1 refers to the set of packets transmitted through *Ro*_1_ and *PT*2 to the set of packets transmitted through *Ro*_2_. Consequently, *PT* = *PT*1 ⋃ *PT*2 and *n*_*pt*_ = |*PT*1| + |*PT*2|.

Once all packets are transmitted, the calculation of the total transmission time on router *Ro*_1_ is denoted by *Tr*_1_ and on router *Ro*_2_ is denoted by *Tr*_2_. Therefore, the maximum estimated transmission time for the two routers is given in Eq 1.

$$\begin{array}{c} Tr_{max}=\text{max}\left(Tr_{1},Tr_{2}\right). \end{array}$$ (1)

**Proposition 1** *The maximum estimated transmission time for the two routers* (*Tr*_*max*_) *can be written as detailed in* Eq 2.

$$\begin{array}{c} Tr_{max}=\text{max}\left(\max_{Pt_{j}\in PT1}\left(Tc_{j}^{1}\right),\underset{Pt_{j}\in PT2}{\text{max}}\left(Tc_{j}^{2}\right)\right). \end{array}$$ (2)

**Proof 1** *The cumulative transmission time of the packet j (finishing transmission time of packet j) on the first router Ro*_1_ *is* $Tc_{j}^{1}$ *and on the second router Ro*_2_ *is* $Tc_{j}^{2}$. *The maximum finishing transmission time for all packets in router Ro*_1_ *is* $\underset{Pt_{j}\in PT1}{\text{max}}\left(Tc_{j}^{1}\right)$. *The maximum finishing transmission time for all packets in router Ro*_2_ *is* $\underset{Pt_{j}\in PT2}{\text{max}}\left(Tc_{j}^{2}\right)$. *The total transmission time on router Ro*_1_ *is Tr*_1_ *and on router Ro*_2_ *is Tr*_2_. *Thus*, $Tr_{1}=\underset{Pt_{j}\in PT1}{\text{max}}\left(Tc_{j}^{1}\right)$ *and* $Tr_{2}=\underset{Pt_{j}\in PT2}{\text{max}}\left(Tc_{j}^{2}\right)$.

One packet cannot be simultaneously transmitted through the two routers in a fixed time. The objective is to give an algorithm that can minimize the value *Tr*_*max*_ respecting the proposed confidentiality constraint presented in this paper. This constraint is to enforce packets belonging to the same confidentiality level to be transmitted through routers simultaneously which means that, in a fixed time interval it is not possible to transmit packets that belongs to the same confidentiality level. Indeed, in the same time interval, the transmission of two packets belonging to the same confidentiality level is not allowed.

The problem of the minimization of the maximum estimated transmission time for the two routers under the confidentiality constraint is an NP-Hard problem as proved in [53].

**Example 1** *Suppose that a problem of transmission network with n*_*pt*_ = 15. *The number of confidentiality level n*_*Cl*_ *is equal to 3. The estimated transmission time tp*_*j*_ *for each packet Pt*_*j*_ *is illustrated in* Table 1.

**Table 1:** Distribution of *tp*_*j*_ for each packet *Pt*_*j*_ for Example 1.

*j*	1	2	3	4	5	6	7	8	9	10	11	12	13	14	15
*Clp*_*j*_	1	2	3	3	2	2	2	3	1	1	3	2	3	1	2
*tp*_*j*_	27	2	18	16	4	28	3	5	27	28	6	20	30	5	15

*The confidentiality levels for each packet are given in* Table 2. *It is noticeable that packets Pt*_1_, *Pt*_9_, *Pt*_10_, *and Pt*_14_ *belong to the same confidentiality level Cl*_1_, *which means that these packets must not, in any case, be transmitted simultaneously at the same time on the two routers*.

**Table 2:** Confidentiality levels distribution.

	*Pt*_*j*_					
*Cl*_1_	1	9	10	14		
*Cl*_2_	2	5	6	7	12	15
*Cl*_3_	3	4	8	11	13	

**Fig 1:**
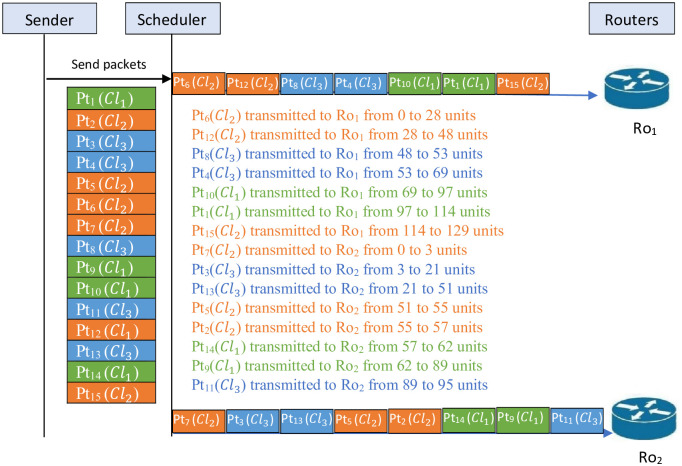
Unfeasible schedule for the studied problem.

*The schedule shown in* Fig 1 *illustrates the scheduling of packets on the two routers without consideration of the confidentiality constraint. This figure shows that Tr*_*max*_ = 129. *The schedule is as follows. The transmitted packets through the router Ro*_1_ *are* {6, 12, 8, 4, 10, 1, 15} *and through the router Ro*_2_ *the transmitted packets are* {7, 3, 13, 5, 2, 14, 9, 11}.

*It is worthy to note that this schedule cannot be considered as a feasible solution for the studied problem. Indeed, a feasible solution to the studied problem must satisfy the confidentiality constraint. Two contradictions of the confidentiality constraint have occurred for the schedule illustrated in* Fig 1. *The packets* {6, 7} *were transmitted in the same interval even though they belong to the same confidentiality level Cl*_2_. *The same holds true with the packets* {9, 10} *which were transmitted in the same interval even though they belong to the same confidentiality level Cl*_1_.

**Fig 2:**
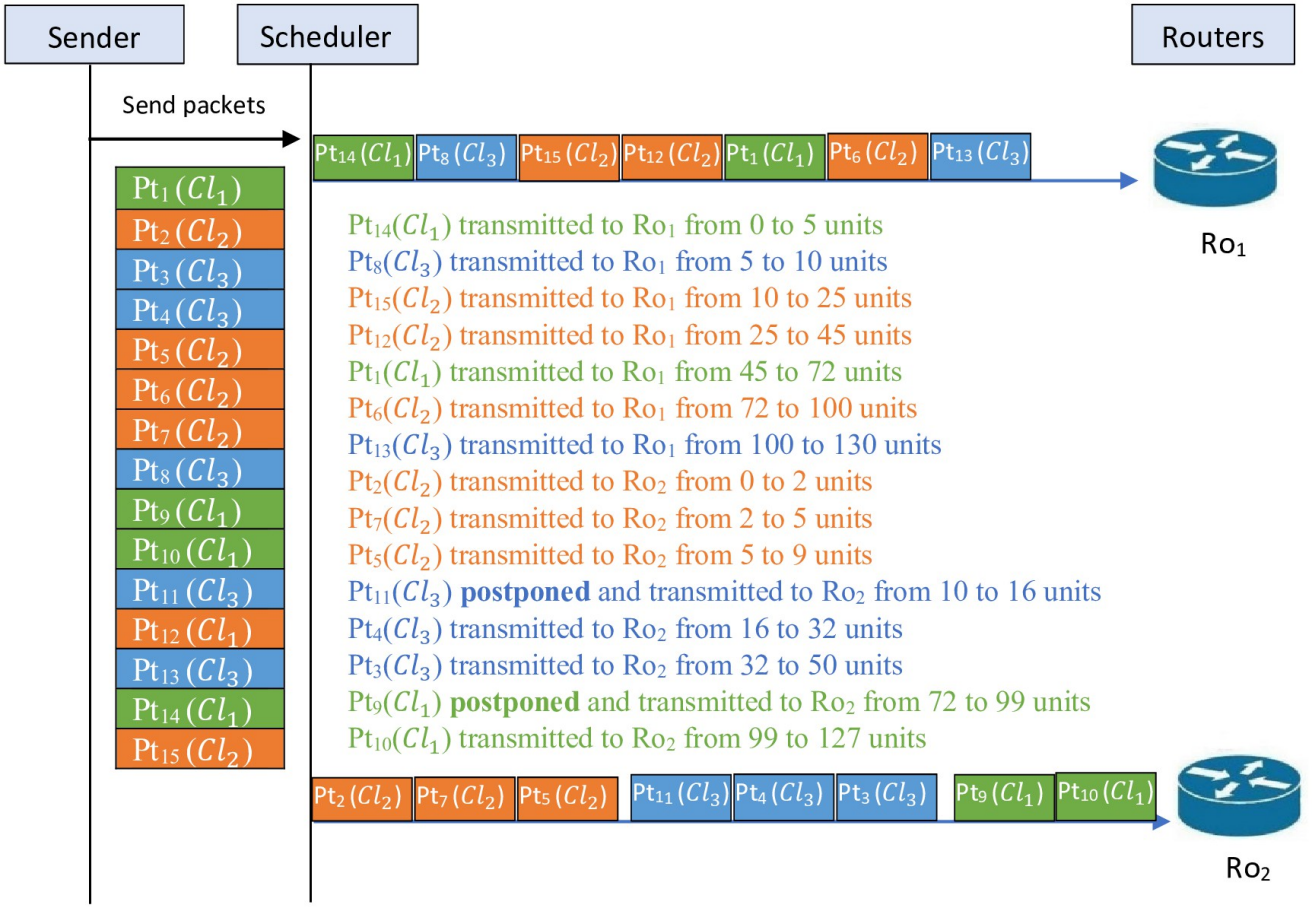
Feasible schedule for the studied problem.

*Now, concerning the constraint proposed in the studied problem, the schedule given in* Fig 1 *cannot be accepted. For this reason, a feasible solution must be presented. In fact, it is illustrated in the schedule shown in* Fig 2. *This figure shows that Tr*_*max*_ = 130. *The schedule is as follows. The packets* {14, 8, 15, 12, 1, 6, 13} *are transmitted through router one Ro*_1_ *and the packets* {2, 7, 5, 11, 4, 3, 9, 10} *are transmitted through router two Ro*_2_.

## 4 Proposed architecture and components

This section details a novel architecture of data transmission into the network. The important advantage of this architecture is adding the component called “scheduler” into the well-known architecture. In addition, a new constraint called “confidentiality constraint” is imposed for the transmission of packets. This constraint precises the confidentiality level required by the administrator or the intelligencer (i.e., an intelligencer is a person that has a very high priority to manage and control the network and each data transmitted through the network. In addition, the intelligencer is responsible for attributing the confidentiality level to each file. As a result, all packets constituting the file will have the same confidentiality level).

We present the following example of the confidentiality levels to clarify the proposed idea.

- *Cl*_1_: Very restricted. This level can encompass all highly sensitive data (files) that can affect national or military security.
- *Cl*_2_: Restricted. This level can encompass all sensitive data (files).
- *Cl*_3_: Confidential. This level can encompass all delicate data (files).
- *Cl*_4_: Internal. This level can encompass all non-sensitive data (files) that can’t be disclosed to the public.
- *Cl*_5_: Public. This level can encompass all revealed data (files) that may be exposed to the public.

Each confidentiality level *Cl*_*i*_ encompasses a fixed number of packets. This number is denoted by *np*_*i*_ ∀*i* ∈ {1, ⋯, *n*_*Cl*_}.

**Proposition 2** *The total number of packets is given in* Eq 3.

$$\begin{array}{c} n_{pt}=\sum_{i=1}^{n_{Cl}}np_{i} \end{array}$$ (3)

**Proof 2** *The sum of all packets in each confidentiality level is the total packets n*_*pt*_.

**Fig 3:**
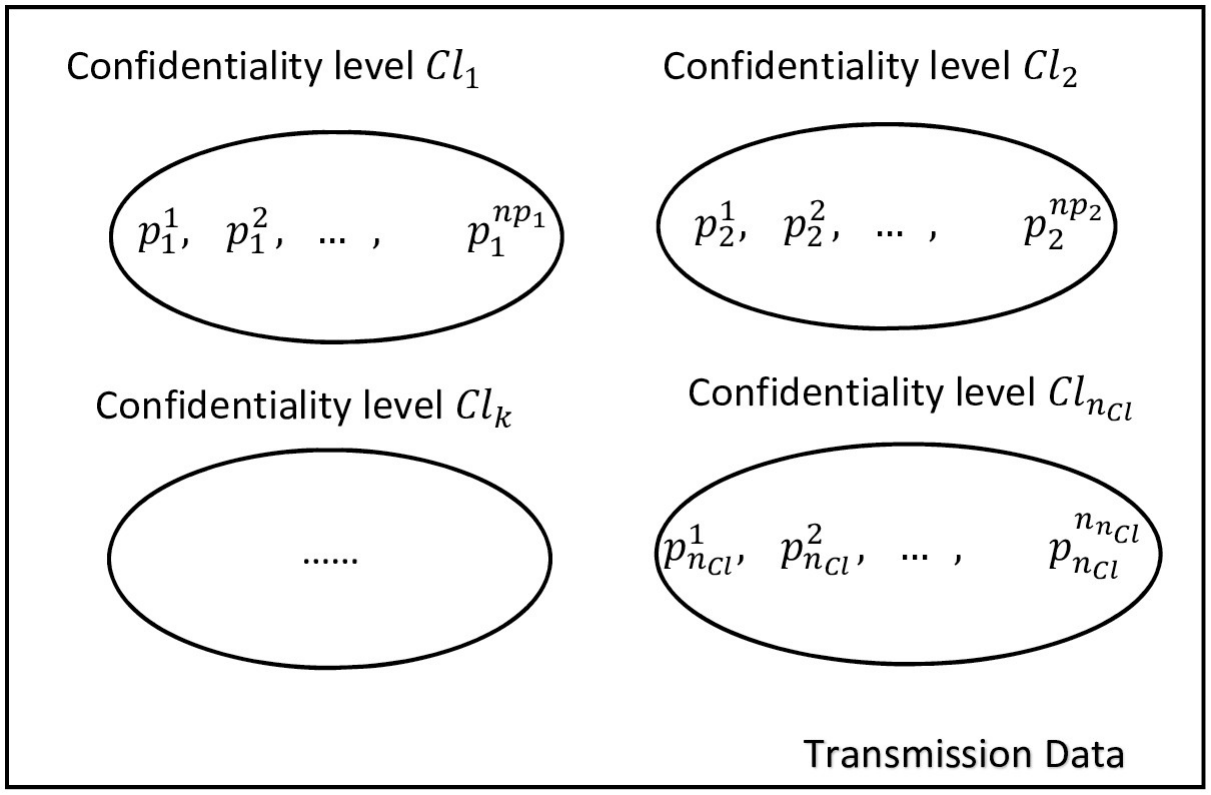
Confidentiality level categorization.

Fig 3 shows the confidentiality level categorization. The confidentiality level *Cl*_*i*_ has a set of packets $\left\{P_{i}^{1},\cdots ,P_{i}^{n_{i}}\right\}$ ∀ *i* ∈ {1, ⋯, *n*_*Cl*_}.

**Fig 4:**
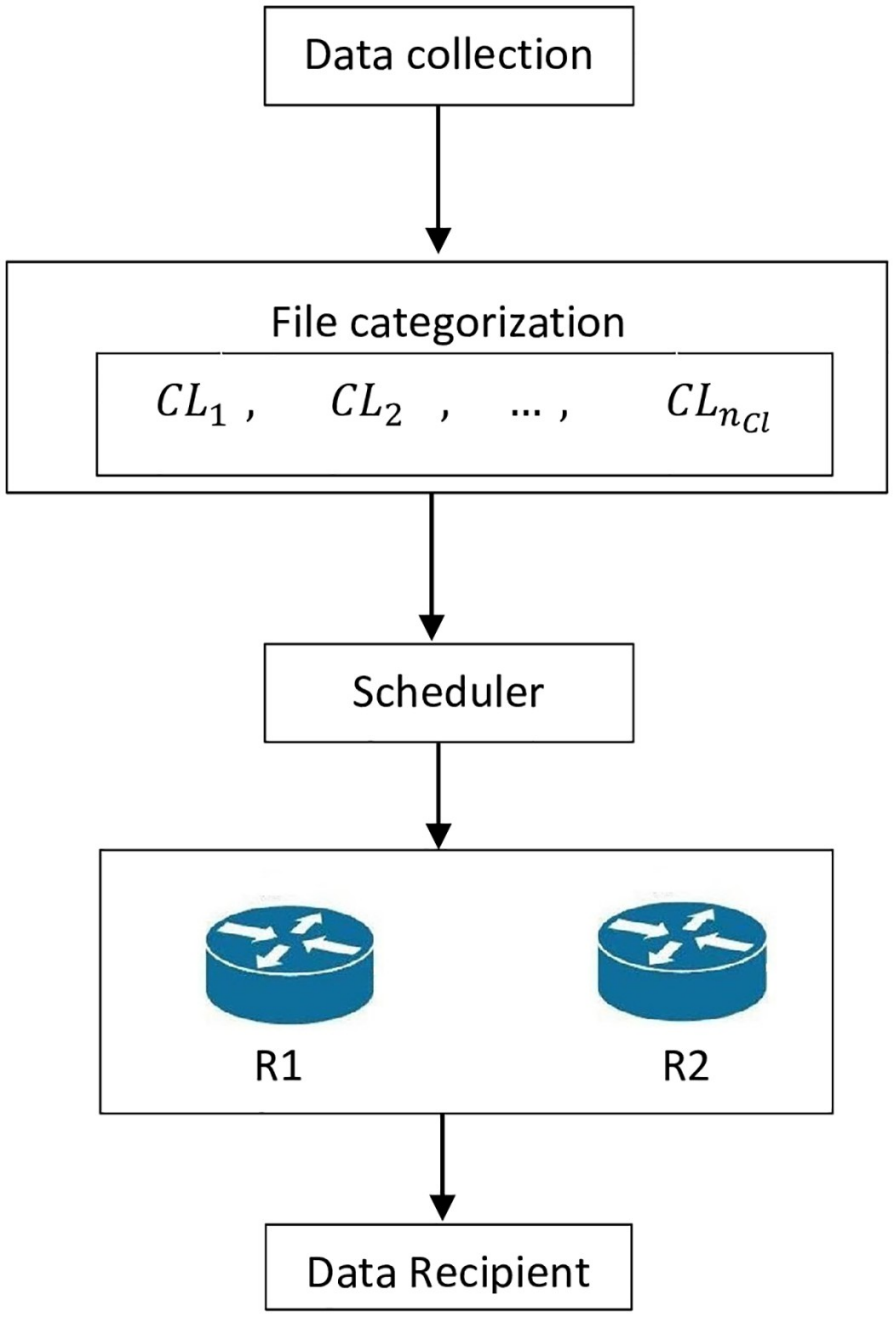
Novel architecture with two routers.

The novel architecture with two routers that can show the addition of the new component “Scheduler” is illustrated in Fig 4. The proposed architecture is composed of five components. These components are described as follows.

- *Data collection*: This component is responsible for specifying the files to be sent. It is managed by the intelligencer of the network.
- *File categorization*: This component is responsible for specifying the confidentiality level. The intelligencer specifies the confidentiality level for each file.
- *Scheduler*: This component is responsible for solving a scheduling problem for the transmission of packets through the network. It is managed or represented by a scheduler, and the solution to the scheduling problem is selected after running several algorithms, and the best solution will be selected.
- *Routers*: Two routers are the entities of this component.
- *Data Recipient*: This component is represented by the receiver of the data.

The novel proposed architecture illustrated in Fig 4 allows manipulating the dispersion and transmission of data.

## 5 Lifting procedure (*LP*)

This section describes a new proposed lifting procedure that can be applied to any given algorithm for the studied problem. This application can enhance the algorithm and gives a better result. The idea of this lifting procedure is as follows. Given an algorithm *A* that solves approximately the studied problem-we denote by *Ro*_*ma*_ the router that has the maximum *Tr*_*k*_ with *k* = {1, 2}. We denote by *Ro*_*mi*_ the router that has the minimum *Tr*_*k*_. The lifting procedure role is to search the first packet scheduled in *Ro*_*ma*_ and remove it to *Ro*_*mi*_ at the last position to be the last packet scheduled in *Ro*_*mi*_. This gives a new schedule for the studied problem. In fact, a new value of *Tr*_*max*_ must be recalculated, and the best solution must be chosen.

**Example 2** *Assume that the number of packets n*_*pt*_ = 15 *and the number of confidentiality levels n*_*Cl*=3_. Table 3 *presents the distribution of tp*_*j*_ *for each packet Pt*_*j*_. *This example shows the resulting schedule before and after applying the lifting procedure*.

**Table 3:** Distribution of *tp*_*j*_ for each packet *Pt*_*j*_ for Example 2.

*j*	1	2	3	4	5	6	7	8	9	10	11	12	13	14	15
*Clp*_*j*_	1	2	3	2	1	1	2	2	2	3	1	3	2	2	2
*tp*_*j*_	21	24	16	30	11	30	13	18	27	17	21	24	27	10	12

**Fig 5:**
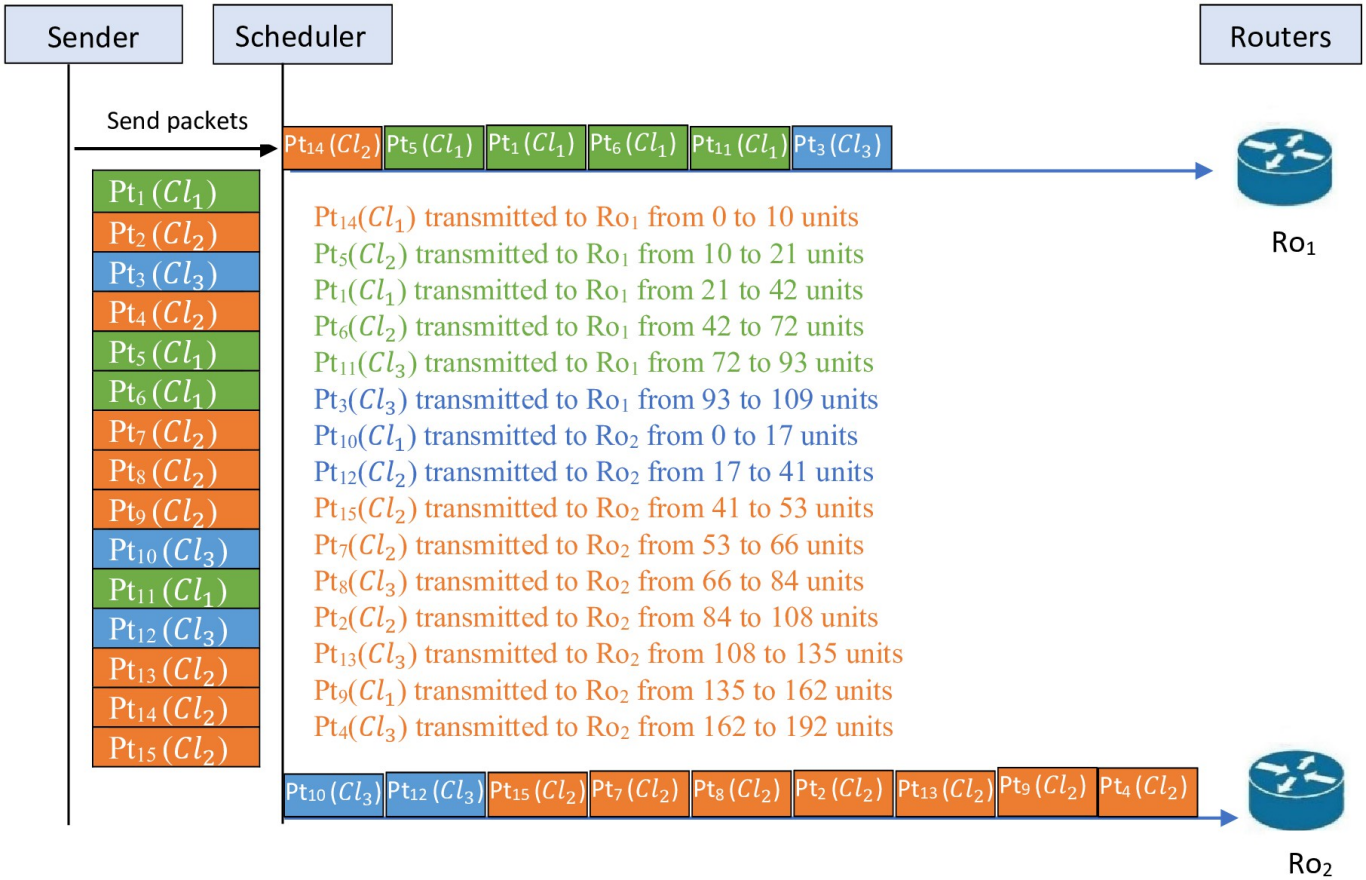
Schedule before applying the lifting procedure for Example 3.

Fig 5 *illustrates the schedule before applying the lifting procedure for Example 3. This figure shows that in Ro*_1_ *the packets* {14, 5, 1, 6, 11, 3} *are transmitted and in Ro*_2_ *the packets* {10, 12, 15, 7, 8, 2, 13, 9, 4} *are transmitted. For this schedule Tr*_*max*_ = 192.

**Fig 6:**
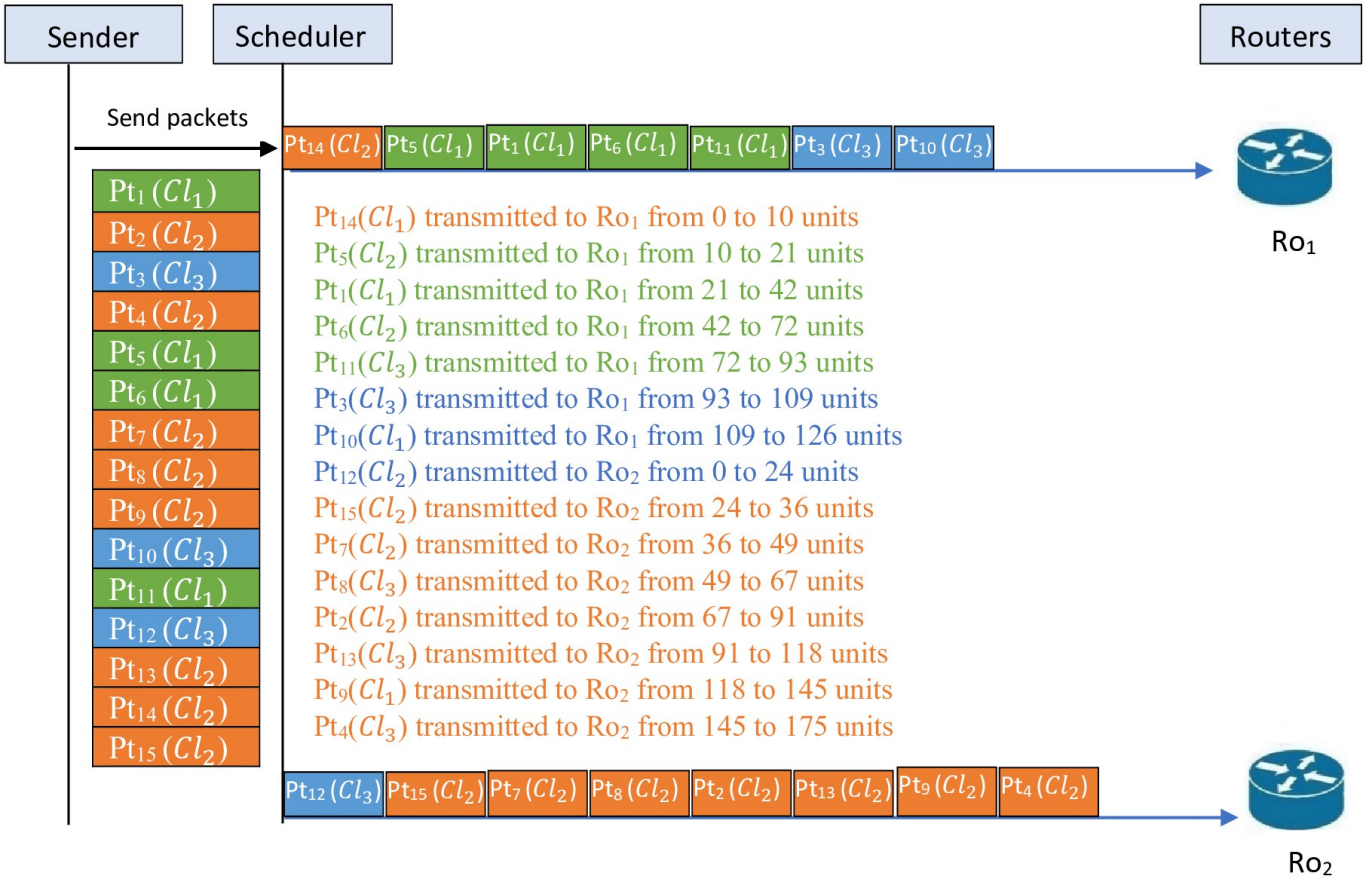
Schedule after applying the lifting procedure for Example 3.

Fig 6 *illustrates the schedule after applying the lifting procedure in Example 3. This figure shows that the packets* {14, 5, 1, 6, 11, 3, 10} *are transmitted through Ro*_1_ *and the packets* {12, 15, 7, 8, 2, 13, 9, 4} *are transmitted through Ro*_2_. *For this schedule Tr*_*max*_ = 175.

*It is clear from* Figs 5 and 6 *that the lifting procedure gives a better remarkable result. Indeed, for the schedule before the using lifting procedure of this example, the total time is Tr*_*max*_ = 192 *compared to the result after applying the lifting procedure, which is equal to Tr*_*max*_ = 175. *So, the difference is 17 units*.

## 6 Proposed algorithms

This section presents detailed instructions to show the functionality of twelve algorithms developed to solve the studied problem. The algorithms are based on the dispatching rules, the local insertion search, the randomization method, and the proposed lifting procedure. The first and second algorithms are based on the dispatching rules using the non-decreasing order and the non-increasing order algorithm. The third algorithm uses a local insertion search approach, and the fourth and the fifth algorithms use a randomization method. The sixth algorithm is based on the critical confidentiality level in which detailed information is presented about the critical confidentiality level. Finally, the six remaining algorithms are lifting of all six presented algorithms.

### 6.1 Longest transmission time first algorithm (*LTF*)

This algorithm is based on selecting the packet that has the longest estimated transmission time. The selected packet will be transmitted through the most available router. The transmission which is-based on the latter selection is continued until all packets are transmitted.

**Proposition 3** *The complexity of the LTF algorithm is O*(*nlogn*).

**Proof 3** *To sort packets, a Quicksort algorithm is applied. As known, Quicksort’s time complexity is O*(*nlogn*). *After that, each packet is assigned to a selected router which takes O*(*n*) *operations for all packets. So, the LTF’s time complexity is O*(*nlogn*).

Example 3 describes the scheduling of the packets on the two routers following *LTF* algorithm.

**Example 3** *Assume that the number of packets is n*_*pt*_ = 8 *and the number of confidentiality levels is n*_*Cl*_ = 3.

**Fig 7:**
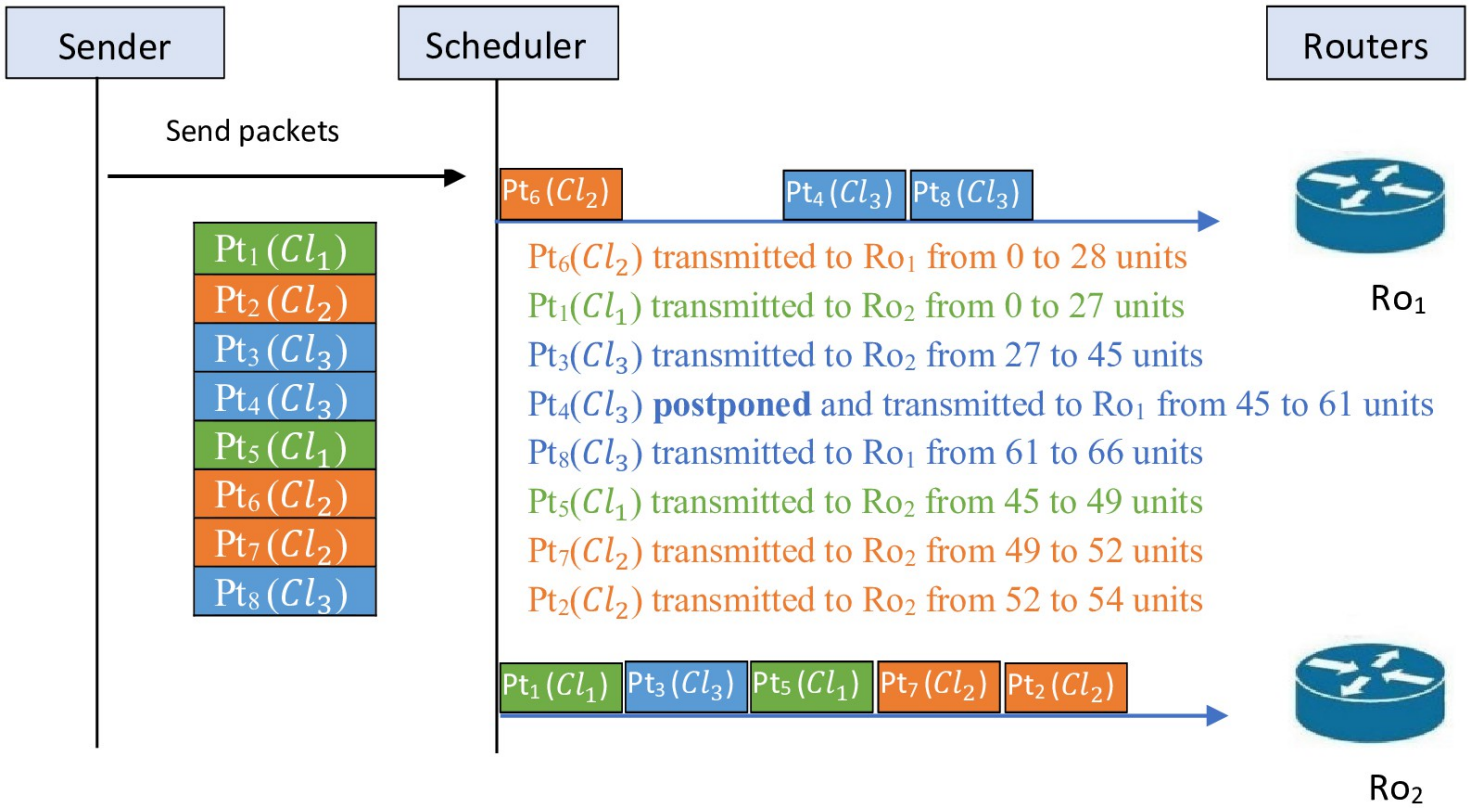
Schedule of *LTF* algorithm.

Fig 7 *illustrated the obtained schedule after applying LTF algorithm. The obtained sequences after applying LTF are* {6, 4, 8} *on Ro*_1_ *and* {1, 3, 5, 7, 2} *on* *Ro*_2_. *This schedule gives the values tr*_1_ = 66 *and tr*_2_ = 54 *with a total estimated transmission time Tr*_*max*_ *value of 66*.

### 6.2 The smallest transmission time first algorithm (*STF*)

This algorithm is based on selecting the packet that has the Smallest estimated transmission time. The selected packet will be transmitted through the most available router. The transmission based on the latter selection is continued until all packets are transmitted.

**Proposition 4** *The complexity of the STF algorithm is O*(*nlogn*).

**Proof 4** *To sort packets, a Quicksort algorithm is applied. As known, Quicksort’s time complexity is O*(*nlogn*). *After that, each packet is assigned to a selected router which takes O*(*n*) *operations for all packets. So, the STF’s time complexity is O*(*nlogn*).

Example 4 describes the scheduling of the packets on the two routers following *STF* algorithm.

**Fig 8:**
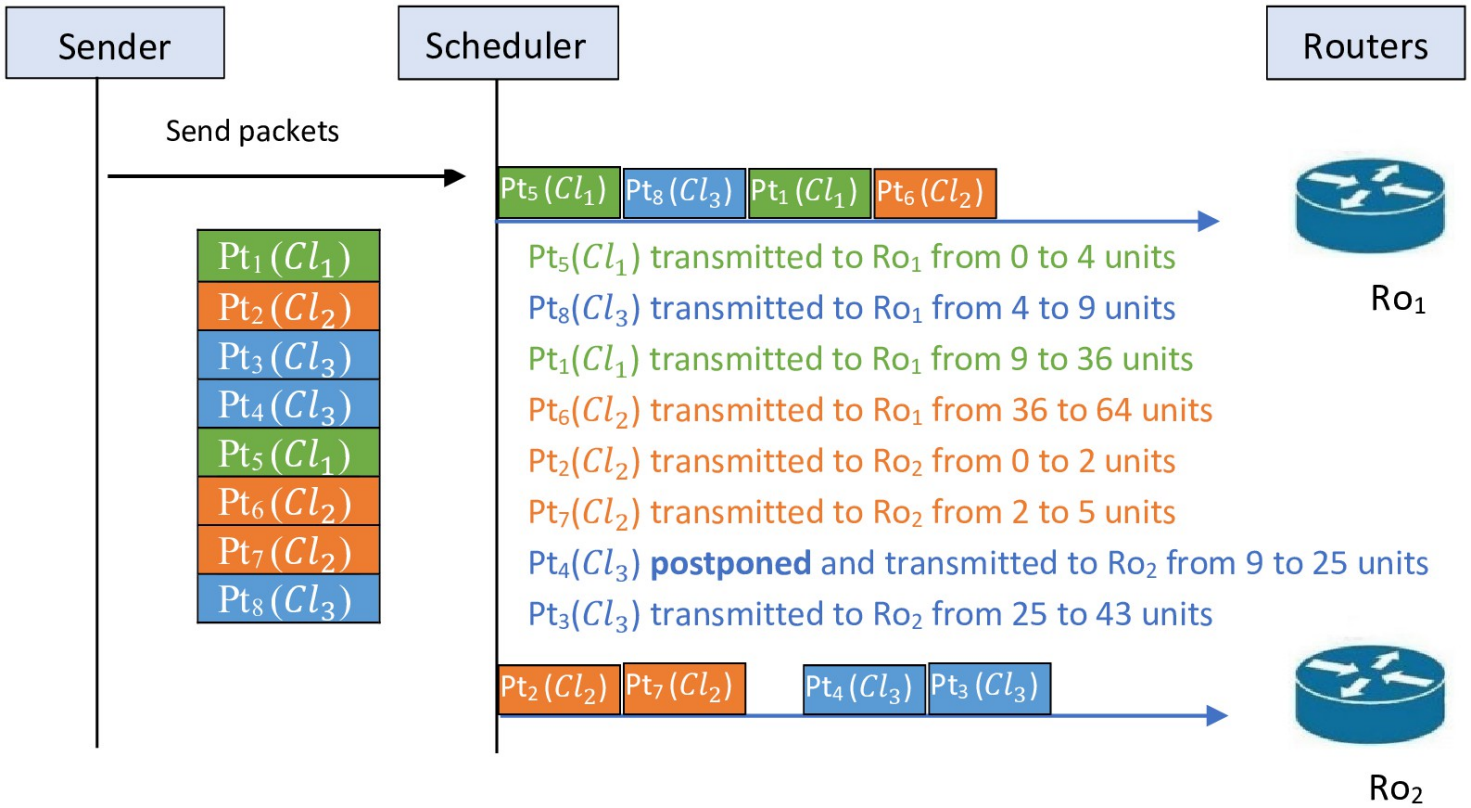
Schedule of *STF* algorithm.

**Example 4** *For this example, we choose to apply the STF algorithm in the example detailed in* Table 4. Fig 8 *shows the obtained schedule after applying STF algorithm. The obtained sequences after applying STF is* {5, 8, 1, 6} *on Ro*_1_ *and* {2, 7, 4, 3} *on Ro*_2_ *this given a total transmission time Tr*_*max*_ *value of 64*.

**Table 4:** Estimated transmission time for each packet *pt*_*j*_ and confidentiality level distribution for *LTF* example.

*j*	1	2	3	4	5	6	7	8
*Clp*_*j*_	27	2	18	16	4	28	3	5
*tp*_*j*_	1	2	3	3	1	2	2	3

*Based on Example 3 and 4, the results are Tr*_*max*_ = 66 *and Tr*_*max*_ = 64 *by applying LTF algorithm and STF algorithm, respectively. Therefore, for the instances in* Table 4, *the result obtained by STF is better than the one obtained by LTF. This remark is not general. This means that the result cannot be the same for all the instances*.

### 6.3 Search, test, and insert algorithm (*STI*)

First, we have to introduce the definition of the idle-time in a schedule.

**Definition 1** *The idle-time is a slot time or interval time when there is no packet transmission*.

Based on the definition-above, this time interval is not exploited and can give a bad result for the scheduling problem on routers. Therefore, in this subsection, the algorithm tends to avoid idle-time and minimize the number of idle-time in routers by inserting packets in these time intervals. Hereinafter, the set of idle-time is denoted by *IT*_1_ and *IT*_2_ in the first router and the second one, respectively. *ni*_1_ and *ni*_2_ denote the number of the idle-time in *Ro*_1_ and *Ro*_2_, respectively. Therefore, $IT_{1}=\left\{IT_{1}^{1},\cdots ,IT_{1}^{ni_{1}}\right\}$ and $IT_{2}=\left\{IT_{2}^{1},\cdots ,IT_{2}^{ni_{2}}\right\}$.

The *SI* algorithm’s first step is to choose the type of sort order of the packets. There are two types of sort orders packets available to be applied. The first one is the non-decreasing order of the estimated transmission time of the packets, and the second is the non-increasing order of the estimated transmission time of the packets.

The first idle-time $IT_{2}^{1}$ or $IT_{1}^{1}$ is obtained after the start of scheduling the packet on the two routers. *pt*_*j*_ must be tested to insert an unscheduled packet in the obtained idle-time. If there is no possibility to schedule any unscheduled packet, the packet *pt*_*j*_ is scheduled, and an idle-time is provoked. Then, the same instructions are called until all packets are scheduled.

Hereinafter, the functions that sort the elements of a given list *Li* in the increasing order and decreasing order are denoted by *InS*() and *DcS*(), respectively.

In algorithm 1, we present an illustration of the search and the insertion packets in the idle-times when the *InS* is applied (*SIPI* procedure).

We denote *SDI*() for the function that searches and determines the idle-times when a packet *pt*_*j*_ is selected to be assigned. This function updates the *ni*_2_ and *ni*_1_ values.

We denote *ST*() for the function that returns the starting time of the obtained idle-times for the scheduled packets.

We denote *PIS*(*r*) for the function that searches the possibility of scheduling the packet *pt*_*j*_ on the router *r*. This function returns 1 if it is possible to schedule the packet *pt*_*j*_, otherwise it returns 0.

**Algorithm 1** Procedure (*SIPI*)

1: Call *InS*(*PT*)

2: Set *ni*_1_ = 0 and *ni*_2_ = 0

3: **for** (*j* = 1 to *n*_*pt*_) **do**

4:  Set *test*1 = 0 and *test*2 = 0

5:  Call *SDI*()

6:  **for** (*k* = 1 to *ni*_1_) **do**

7:   Call *ST*()

8:   **if** (*PIS*(1) = 1) **then**

9:    Set *test*1++

10:    calculate $Tc_{j}^{1}$

11:   **end if**

12:  **end for**

13:  **for** (*k* = 1 to *ni*_2_) **do**

14:   Call *ST*()

15:   **if** (*PIS*(2) = 1) **then**

16:    Set *test*2++

17:    calculate $Tc_{j}^{2}$

18:   **end if**

19:  **end for**

20:  **if** (*test*1 ≠ 0 OR *test*2 ≠ 0) **then**

21:   $Tc_{j}=\text{min}\left(Tc_{j}^{1},Tc_{j}^{2}\right)$

22:  **end if**

23: **end for**

24: Calculate *Tr*_*max*_

25: Return *Tr*_*max*_

The *SIPI* procedure is functioning as follows. Firstly, we call the procedure *InS*() applied on all packets. The numbers of idle-times *ni*_1_ and *ni*_2_ are initialized to 0. A loop from 1 to the number of packets is applied to schedule packets in *Ro*_1_ and *Ro*_2_. This loop is described from instruction 3 to 23. For each packet (for each value of *j*), we Call *SDI*() (Instruction 5) and we loop from 1 to the number of idle-times in *Ro*_1_ trying to insert the packet *j*. If the insertion can be feasible, so we calculate $TC_{j}^{1}$, otherwise a test for the next idle-time will take place (Instructions 6–12). After that, we loop from 1 to the number of idle-times in *Ro*_2_ trying to insert the packet *j*. If the insertion can be feasible, so we calculate $TC_{j}^{2}$, otherwise a test for the next idle-time will take place (Instructions 13–19). Finally, the minimum cumulative time will be picked (Instructions 20–22).

The algorithm of the search and the insertion of the packets in the idle-times when the *DcS* is applied uses the same instructions in Algorithm 1, but it replaces *InS*(*PT*) by *DcS*(*PT*). This algorithm is denoted by *SIPD*()

Now, the instructions of the search and insert algorithm (*STI*) are illustrated in Algorithm 2.

**Algorithm 2** Search and insert algorithm (*STI*)

1: Set *C*_1_ = *SIPI*(*PT*)

2: Set *C*_2_ = *SIPD*(*PT*)

3: Determine *Tr*_*max*_ = min(*C*_1_, *C*_2_)

4: Return *Tr*_*max*_

The Search and insert algorithm (*STI*) is functioning as follows. We call *SIPI*(*PT*) and *SIPD*(*PT*). The best value is picked.

**Proposition 5** *The complexity of the STI algorithm is O*(*n*^2^).

**Proof 5** *To sort packets, a Quicksort algorithm is applied. So InS*(*PT*)’*s and DcS*(*PT*)’*s time complexities are O*(*nlogn*). *Then, the SDI*() *function takes n*^2^ *comparisons so that a quadratic complexity O*(*n*^2^). *The ST*() *function has constant complexity. Therefore, SIPI*(*PT*)’*s time complexity is O*(*n*^2^) *and the same for SIPD*(*PT*) *algorithm. So overall, the STI*’*s time complexity is O*(*n*^2^).

### 6.4 Randomized Longest Transmission time first algorithm (*RLT*)

Firstly, we sort all packets according to the decreasing order of their estimated transmission time. Now, we schedule the first-longest estimated transmission time with probability *β* and the second-longest estimated transmission time with probability 1 − *β*. In practice, the value of *β* is equal to 0.3.

We denote *SWP*() for the procedure that swaps two packets given as inputs.

The instructions described in Algorithm 3 show the functionality of *RLT* algorithm.

**Algorithm 3** Algorithm (*RLT*)

1: Call *DcS*(*PT*)

2: **for** (*it* = 1 to 500) **do**

3:  **for** (*j* = 1 to *n*_*pt*_ − 1) **do**

4:   *x* = 1 + rand()%100.

5:   **if**(*x* < 30) **then**

6:    *SWP*(*PT*, *j*, *j* + 1)

7:   **end if**

8:   calculate $Tc_{j}^{1}$

9:   calculate $Tc_{j}^{2}$

10:   **if** ($Tc_{j}^{1}<Tc_{j}^{2}$) **then**

11:    *Ro*(*j*) = *Ro*_1_

12:   **else**

13:    **if** ($Tc_{j}^{1}>Tc_{j}^{2}$) **then**

14:     *Ro*(*j*) = *Ro*_2_

15:    **end if**

16:   **end if**

17:   Calculate *Tc*_*j*_

18:  **end for**

19:  **if** (*j* = *n*_*pt*_) **then**

20:   Set *j*++

21:   Goto Step 8

22:  **end if**

23:  Calculate $Tr_{max}^{it}$

24: **end for**

25: Calculate $Tr_{max}=\underset{1\leq it\leq 500}{\text{min}}Tr_{max}^{it}$

26: Return *Tr*_*max*_

The randomized longest transmission time first algorithm (*RLT*) is functioning as follows. Firstly, we call *DcS*(*PT*) (Instruction 1) to sort all packets according to the decreasing order of their estimated transmission time. After that, an iterative loop is applied 500 times (Instruction 2) to randomly choose the packet that will be scheduled. Indeed, for each iteration, a loop of *n*_*pt*_ − 1 will take place (Instruction 3) and for each packet, a random generation of a number between 1 and 100 will be applied (Instruction 4). If this number is less than 30 (Instruction 5) then we swap the packets *j* and *j* + 1 (Instruction 6). We schedule the selected packet on the most available router (Instructions 8–17). Next, the last remaining packet will be scheduled (Instructions 19–21). After that, $Tr_{max}^{it}$ is calculated (Instruction 23). After the termination of all iterations, the minimum value of *Tr*_*max*_ is determined (Instruction 25).

**Proposition 6** *The complexity of the RLT algorithm is O*(*nlogn*).

**Proof 6** *As mentioned above, the Quicksort algorithm is used to sort packets. So DcS*(*PT*)’*s time complexity is O*(*nlogn*). *Then, each packet is assigned to a selected router which takes n operations. Since the randomize function has a constant complexity, the RLT*’*s time complexity is O*(*nlogn*).

### 6.5 Randomized smallest Transmission time first algorithm (*RST*)

This algorithm is like *RLT* but instead of *DcS*(*PT*) we use *InS*(*PT*). Then, the *RST*’s complexity time is *O*(*nlogn*).

### 6.6 Critical confidentiality level algorithm (*CCL*)

Firstly, we define the critical confidentiality level. The packets having the same confidentiality level *Cl*_*i*_ are grouped in a set denoted by *PT*_*i*_ with *i* = {1, ⋯, *n*_*Cl*_}. The sum of the estimated transmission time of all packets in *PT*_*i*_ is denoted by *St*_*i*_.

**Definition 2** *The critical confidentiality level denoted by C is the confidentiality level that has the maximum St*_*i*_ ∀*i* = {1, ⋯, *n*_*Cl*_}.

**Definition 3** *The fictive packet denoted by FP*_*i*_ *is a new proposed packet with the estimated transmission time St*_*i*_ ∀*i* = {1, ⋯, *n*_*Cl*_}.

**Proposition 7** *The number of the fictive packet is n*_*Cl*_.

**Example 5** *Suppose that the estimated transmission time and the confidentiality levels distribution for each packet are the same given in* Table 1.

Table 5 *illustrated the generation of the fictive packets. The estimated transmission time of the fictive packets FP*_1_, *FP*_2_, *and FP*_3_ *are 87, 72 and 75 respectively*.

**Table 5:** The fictive packets regrouping the originally packets.

	*j*					
*FP*_1_	1	9	10	14		
*FP*_2_	2	5	6	7	12	15
*FP*_3_	3	4	8	11	13	

Hereinafter, the function *DFP*() is developed to calculate the total estimated time of each confidentiality level and to determine the *FPs*.

The instructions of *CCL* are illustrated in Algorithm 4. The complexity of this algorithm is *O*(*nlogn*).

**Algorithm 4** Critical confidentiality level algorithm (*CCL*)

1: Call *DFP*(*PT*, *Cl*, *n*_*Cl*_)

2: Call *DcS*(*FP*)

3: **for** (*k* = 1 to *n*_*Cl*_) **do**

4:  Calculate $Tc_{FP_{k}}^{1}$

5:  Calculate $Tc_{FP_{k}}^{2}$

6:  $Tc_{FP_{k}}=\text{min}\left(Tc_{FP_{k}}^{1},Tc_{FP_{k}}^{2}\right)$

7: **end for**

8: Calculate $Tr_{max}=\underset{1\leq k\leq n_{Cl}}{\text{max}}Tc_{FP_{k}}$

9: Return *Tr*_*max*_

The critical confidentiality level algorithm (*CCL*) is functioning as follows. Firstly, we call *DFP*() and *DcS* (Instructions 1–2). After that, a loop from 1 to the number of confidentiality level will take place to determine $Tc_{FP_{k}}^{1}$ and $Tc_{FP_{k}}^{2}$ (Instructions 4–5). The minimum value will be stored in $Tc_{FP_{k}}$ (Instruction 6). Finally, *Tr*_*max*_ is calculated (Instruction 8).

**Proposition 8** *The complexity of the CCL algorithm is O*(*nlogn*).

**Proof 7** *The complexity of DFP function is O*(*n*). *As mentioned above, the Quicksort algorithm is used to sort packets. So DcS*(*FP*)’*s time complexity is O*(*nlogn*). *Then, each packet is assigned to the most available router which takes O*(*n*) *operations. So, the CCL*’*s time complexity is O*(*nlogn*).

### 6.7 Lifting algorithms

All algorithms proposed previously can be enhanced by applying the lifting procedure *LP* defined in Section 5. The lifting algorithm of *LTF*, *STF*, *STI*, *RLT*, *RST*, and *CCL* will be $\bar{LTF}$, $\bar{STF}$, $\bar{STI}$, $\bar{RLT}$, $\bar{RST}$ and $\bar{CCL}$, respectively. Since *LP*’s time complexity is *O*(*n*), then all lifting algorithms maintain the same complexity class proven above.

## 7 Experimental results

The experimental results and the statistical analyses are detailed in this section. The proposed algorithms are coded in C++. The computer running all developed programs has the characteristics for the processor Intel(R) Core (TM) i5–1035G1CPU @1.00GHz and for the memory 8GB RAM.

### 7.1 Problem instances

To measure the performance of the proposed algorithms, it is mandatory to test them on several instances. The different classes of instances depend on how the generation of the estimated transmission time *tp*_*j*_ will be. In this paper, we opted for the uniform distribution to generate all instances. This distribution is denoted by *U*[*s*, *e*], with *s* being the starting value of *tp*_*j*_, and *e* the ending value that can’t be exceeded. Four different classes are developed as follows:

- *Class* A: *tp*_*j*_ in *U*[1, 30].
- *Class* B: *tp*_*j*_ in *U*[10, 30].
- *Class* C: *tp*_*j*_ in *U*[1, 50].
- *Class* D: *tp*_*j*_ in *U*[20, 50].

The number of packets *n*_*pt*_ is varying in {15, 25, 45, 65, 90, 110}. The number of confidentiality level *n*_*Cl*_ is varying in {3, 5, 7, 9}. For each *n*_*pt*_ value all *n*_*Cl*_ values are tested. For each *n*_*pt*_, *n*_*Cl*_, and *Class*, 10 instances are given. In total (6 × 4 × 4 × 10) = 960 instances are generated and tested. It is worthy noting that the confidentiality level *Clp*_*j*_ for a packet *pt*_*j*_ is generated uniformly between [1, *Clp*_*j*_].

### 7.2 Metrics

The experimental study will only be important after having measured the performance via measurement metrics in order to be able to interpret them afterward. The metrics used in this paper to measure the performance of the proposed algorithms are as follows:

- *A*_+_: best returned value of the *Tr*_*max*_ after the execution of all algorithms.
- *A*: returned value of *Tr*_*max*_ by the studied algorithm.
- *Pr*: percentage of instances when *A*_+_ = *A*.
-
$Gp=\frac{A-A_{+}}{A_{+}}$ .
- *AvG*: average of *Gp* for a group of instances.
- *AT*: average execution time in seconds. When this time is less than 0.001 s the symbol “-” is placed.

### 7.3 Discussions

In this subsection, a discussion of the experimental results is presented. Firstly, an overview of *Pr*, *AvG*, and *AT* for all proposed algorithms is described. Next, the impact of applying the lifting procedure is illustrated. A comparison of the average gap *AvG* for all algorithms according to the number of packets *n*_*pt*_, *n*_*Cl*_, and *Class* is discussed.

Table 6 presents the overview of *Pr*, *AvG*, and *AT* for all proposed algorithms. It shows that the best algorithm that gives the highest percentage of 73.5% is $\bar{RLT}$. The average obtained gap for this algorithm is 0.002. It is remarkable in this respect that all the running times for all algorithms are less than 0.001 s. This proves that the proposed algorithms give a result in a very impressive time. The second best algorithm is *RLT* with a percentage of 72.8% and an average gap of 0.003.

**Table 6:** Overview of *Pr*, *AvG*, and *AT* for all proposed algorithms.

	*LTF*	*STF*	*STI*	*RLT*	*RST*	*CCL*	$\bar{LTF}$	$\bar{STF}$	$\bar{STI}$	$\bar{RLT}$	$\bar{RST}$	$\bar{CCL}$
*Pr*	6.0%	0.2%	24.8%	72.8%	18.6%	53.6%	6.6%	0.2%	24.8%	73.5%	18.6%	54.7%
*AvG*	0.038	0.049	0.011	0.003	0.010	0.006	0.024	0.043	0.011	0.002	0.009	0.005
*AT*	-	-	-	-	-	-	-	-	-	-	-	-

Now, we compare the best algorithm given in literature which is *MDETA* as detailed in [53] with the best-proposed algorithm $\bar{RLT}$. We denoted by *MV* the minimum obtained between $\bar{RLT}$ and *MDETA* after running all the 960 instances. The results show that $\bar{RLT}$ reaches *MV* value in 91% of instances, while *MDETA* reaches the *MV* value in only 41% of instances. This proves that the proposed algorithm $\bar{RLT}$ is the best algorithm compared with the results in the literature.

Table 7 presents the impact of applying the lifting procedure. This table shows that the lifting procedure gives a better result for the algorithms *RLT* and *CCL*.

**Table 7:** Impact of applying the lifting procedure in percentage *Pr*.

	Before lifting	After lifting
*LTF*	6.0%	6.6%
*STF*	0.2%	0.2%
*STI*	24.8%	24.8%
*RLT*	72.8%	73.5%
*RST*	18.6%	18.6%
*CCL*	53.6%	54.7%

**Fig 9:**
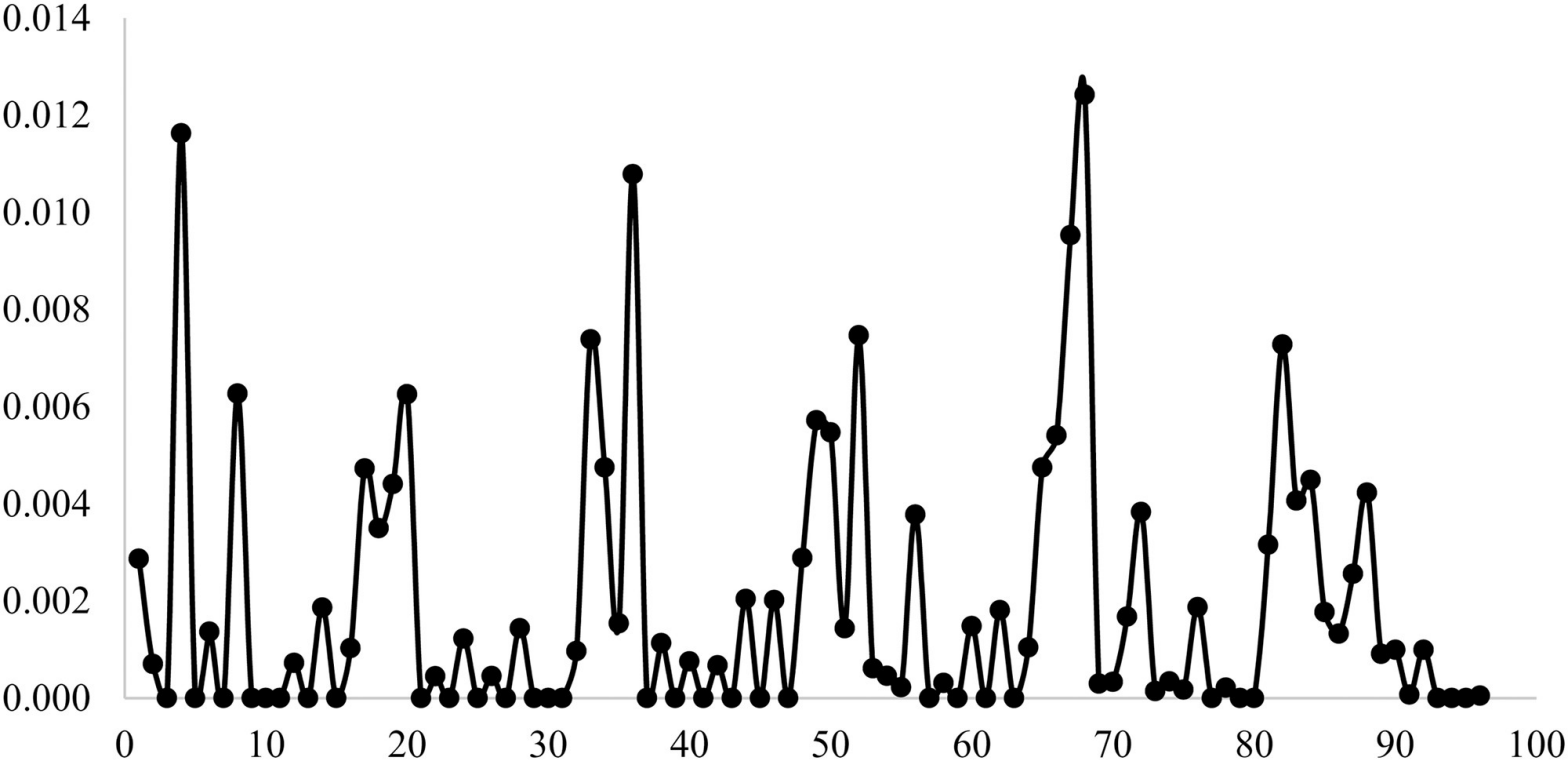
The variation of the average gap values when *Tu* changes for algorithm $\bar{RLT}$.

Each tuple (*n*_*pt*_), *n*_*Cl*_, *Class* is denoted by the variable *Tu*. For the instances given in this experimental results, *Tu* = {1, ⋯, 96}. The first value of *Tu* is 1 and is represented by the tuple (15, 3, 1). The last value of *Tu* is 96 and is represented by the tuple (100, 9, 4). Fig 9 illustrated the variation of the average gap values when *Tu* changes for algorithm $\bar{RLT}$. This figure shows that the remarkable peaks are recorded for *Tu* = {4, 36, 68} which represented the tuples (15, 3, 4), (45, 3, 4), and (90, 3, 4) respectively. The average gap of 0.012 is obtained when *Tu* = {4, 68}. However, when *Tu* = 36 the average gap is equal to 0.011.

Table 8 compares the average gap *AvG* for all algorithms according to the number of packets *n*_*pt*_. It shows that the minimum average gap *AvG* of 0.001 is reached for the algorithm $\bar{RLT}$ when *n*_*pt*_ = 25. However, this minimum *AvG* value is reached for algorithm $\bar{CCL}$ three times when *n*_*pt*_ = {65, 90, 110}. It is noted that, for all algorithms excluding *RLT* and $\bar{RLT}$, the average gap *AvG* decreases when the number of packets *n*_*pt*_ increases. The maximum average gap of 0.093 is obtained by algorithm *STF* when *n*_*pt*_ = 15.

**Table 8:** Comparison of the average gap *AvG* for all algorithms according to the number of packets *n*_*pt*_.

*n*_*pt*_	*LTF*	*STF*	*STI*	*RLT*	*RST*	*CCL*	$\bar{LTF}$	$\bar{STF}$	$\bar{STI}$	$\bar{RLT}$	$\bar{RST}$	$\bar{CCL}$
15	0.045	0.093	0.028	0.003	0.013	0.016	0.036	0.090	0.027	0.002	0.013	0.014
25	0.042	0.072	0.018	0.002	0.011	0.009	0.029	0.066	0.017	0.001	0.010	0.009
45	0.040	0.049	0.009	0.003	0.011	0.004	0.025	0.043	0.008	0.002	0.009	0.004
65	0.036	0.037	0.006	0.002	0.009	0.002	0.021	0.031	0.006	0.002	0.008	0.001
90	0.035	0.025	0.004	0.003	0.009	0.001	0.019	0.017	0.004	0.003	0.007	0.001
110	0.032	0.019	0.002	0.002	0.007	0.001	0.016	0.013	0.002	0.002	0.006	0.001

Table 9 on the other hand, compares the average gap *AvG* for all algorithms according to the number of confidentiality level *n*_*Cl*_. The minimum reached average gap *AvG* of less than 0.001 is recorded for algorithm $\bar{RLT}$ when *n*_*Cl*_ = 9 and for algorithm *RLT* when *n*_*Cl*_ = 9. In addition, the second best average gap *AvG* of 0.001 is reached for algorithm $\bar{RLT}$ when *n*_*Cl*_ = {5, 7} and for the algorithm *RLT* when *n*_*Cl*_ = {5, 7}. The maximum average gap of 0.063 is obtained by the algorithm *STF* when *n*_*Cl*_ = 3.

**Table 9:** Comparison of the average gap *AvG* for all algorithms according to the number of confidentiality level *n*_*Cl*_.

*n*_*Cl*_	*LTF*	*STF*	*STI*	*RLT*	*RST*	*CCL*	$\bar{LTF}$	$\bar{STF}$	$\bar{STI}$	$\bar{RLT}$	$\bar{RST}$	$\bar{CCL}$
3	0.050	0.063	0.017	0.008	0.021	0.007	0.028	0.052	0.015	0.005	0.018	0.007
5	0.044	0.048	0.011	0.001	0.007	0.006	0.027	0.043	0.010	0.001	0.006	0.005
7	0.033	0.046	0.009	0.001	0.007	0.005	0.022	0.041	0.009	0.001	0.006	0.005
9	0.026	0.039	0.008	0.000	0.005	0.004	0.020	0.037	0.008	0.000	0.005	0.004

Table 10 presents the comparison of the average gap *AvG* for all algorithms according to the different classes *Class*. For the best algorithm, $\bar{RLT}$ the minimum average gap of 0.001 is obtained when *Class* = {1, 3}. However, for *Class* 2 the average gap is equal to 0.002, and for *Class* 4 the average gap is equal to 0.004. Finally, the maximum average gap of 0.056 is obtained for *Class* 3 by the algorithm *STF*.

**Table 10:** Comparison of the average gap *AvG* for all algorithms according to the different classes *class*.

*Class*	*LTF*	*STF*	*STI*	*RLT*	*RST*	*CCL*	$\bar{LTF}$	$\bar{STF}$	$\bar{STI}$	$\bar{RLT}$	$\bar{RST}$	$\bar{CCL}$
1	0.034	0.054	0.007	0.001	0.010	0.004	0.023	0.046	0.007	0.001	0.008	0.004
2	0.036	0.046	0.013	0.002	0.011	0.007	0.025	0.040	0.012	0.002	0.009	0.006
3	0.037	0.056	0.009	0.002	0.010	0.005	0.021	0.052	0.009	0.001	0.009	0.005
4	0.046	0.039	0.015	0.004	0.009	0.006	0.028	0.035	0.014	0.004	0.008	0.006

## 8 Conclusion and prospects

### 8.1 Conclusion

This research studied the problem of scheduling multilevel classified network packets on two routers based on a constraint. We proposed an architectural paradigm that can be deployed for a private network. The paradigm can be used for the secure dissemination of classified data in a military-based environment. This is a known NP-hard in the strongest sense.

We proposed six heuristic algorithms and their enhancements versions using the lifting procedure. The proposed heuristics are *LTF*, *STF*, *STI*, *RLT*, *RST*, and *CCL*. So, in total, we proposed twelve algorithms. We compared the proposed algorithms’ average gap based on the number of packets *n*_*pt*_, *n*_*Cl*_, and *Class*. Our observation based on the performed experimentation indicates that $\bar{RLT}$ algorithm performed the best, recording a percentage rate of 73.5%, and an average gap of 0.003. The second best algorithm in line is *RLT*. *RLT* percentage rate was 72.8%, and the average gap was 0.002. The lifting procedure gave the *RLT* and *CCL* algorithms a better result, and the average gap decreases for all algorithms, excluding $\bar{RLT}$, and *RLT*, when the number of packets *n*_*pt*_ increases. We also noticed that the minimum reaches average gap *AvG* of less than 0.001 according to the number of confidentiality level *n*_*Cl*_ was for algorithm *RLT* when *n*_*Cl*_ = 9.

### 8.2 Prospects

Future work will be based mainly on five directives. The first directive is meant to enhance the proposed algorithms using several meta-heuristics by calling the proposed algorithms the initial solution. The second directive is to develop a lower bound and an exact solution for the studied problem compared with the obtained results. The third directive is to elaborate an extension of the studied problem by considering the problem when the number of routers exceeds 2. The fourth directive is to employ supervised machine learning classification to intelligently label and then transmit the network packets through three or more routers. The last directive is the simulation of the proposed network and algorithms in a real-life scenario with demonstration and testing in real hardware and the investigation of multilevel data security using multi-agent systems.

## Supporting information

S1 Data (TXT)

S1 File (TXT)

## References

[1] Pawar MV, Anuradha J. Network security and types of attacks in network. Procedia Computer Science. 2015;48:503–506. doi: 10.1016/j.procs.2015.04.126

[2] Singh S, Jha RK. A survey on software defined networking: Architecture for next generation network. Journal of Network and Systems Management. 2017;25(2):321–374. doi: 10.1007/s10922-016-9393-9

[3] Paul S, Pan J, Jain R. Architectures for the future networks and the next generation Internet: A survey. Computer Communications. 2011;34(1):2–42. doi: 10.1016/j.comcom.2010.08.001

[4] Jang-Jaccard J, Nepal S. A survey of emerging threats in cybersecurity. Journal of Computer and System Sciences. 2014;80(5):973–993. doi: 10.1016/j.jcss.2014.02.005

[5] Almomani O, Al-Shugran M, Alzubi JA, Alzubi OA. Performance evaluation of position-based routing protocols using different mobility models in manet. International Journal of Computer Applications. 2015;119(3). doi: 10.5120/21050-3692

[6] Ponsam JG, Srinivasan R. A survey on MANET security challenges, attacks and its countermeasures. International Journal of Emerging Trends & Technology in Computer Science (IJETTCS). 2014;3(1):274–279.

[7] Aloqaily M, Kanhere S, Bellavista P, Nogueira M. Special Issue on Cybersecurity Management in the Era of AI. Journal of Network and Systems Management. 2022;30(3):1–7. doi: 10.1007/s10922-022-09659-3

[8] Leighton T, Maggs B, Richa AW. Fast algorithms for finding O (congestion+ dilation) packet routing schedules. Combinatorica. 1999;19(3):375–401. doi: 10.1007/s004930050061

[9] Yan M, Lam KY, Han S, Chan E, Chen Q, Fan P, et al. Hypergraph-based data link layer scheduling for reliable packet delivery in wireless sensing and control networks with end-to-end delay constraints. Information Sciences. 2014;278:34–55. doi: 10.1016/j.ins.2014.02.006

[10] Jemmali M, Alquhayz H. Time-slots transmission data algorithms into network. In: 2020 International Conference on Computing and Information Technology (ICCIT-1441). IEEE; 2020. p. 1–4.

[11] Jemmali M, Denden M, Boulila W, Jhaveri RH, Srivastava G, Gadekallu TR. A Novel Model Based on Window-Pass Preferences for Data-Emergency-Aware Scheduling in Computer Networks. IEEE Transactions on Industrial Informatics. 2022. doi: 10.1109/TII.2022.3149896

[12] Alquhayz H, Jemmali M. Fixed Urgent Window Pass for a Wireless Network with User Preferences. Wireless Personal Communications. 2021;120(2):1565–1591. doi: 10.1007/s11277-021-08524-x

[13] Mahjabin T, Xiao Y, Sun G, Jiang W. A survey of distributed denial-of-service attack, prevention, and mitigation techniques. International Journal of Distributed Sensor Networks. 2017;13(12):1550147717741463. doi: 10.1177/1550147717741463

[14] Kayes A, Kalaria R, Sarker IH, Islam M, Watters PA, Ng A, et al. A survey of context-aware access control mechanisms for cloud and fog networks: Taxonomy and open research issues. Sensors. 2020;20(9):2464. doi: 10.3390/s20092464 32349242 PMC7249653

[15] Tseng FH, Chou LD, Chao HC. A survey of black hole attacks in wireless mobile ad hoc networks. Human-centric Computing and Information Sciences. 2011;1(1):1–16. doi: 10.1186/2192-1962-1-4

[16] Bhushan B, Sahoo G. Recent advances in attacks, technical challenges, vulnerabilities and their countermeasures in wireless sensor networks. Wireless Personal Communications. 2018;98(2):2037–2077. doi: 10.1007/s11277-017-4962-0

[17] Dong S, Abbas K, Jain R. A survey on distributed denial of service (DDoS) attacks in SDN and cloud computing environments. IEEE Access. 2019;7:80813–80828. doi: 10.1109/ACCESS.2019.2922196

[18] Qiu S, Liu Q, Zhou S, Wu C. Review of artificial intelligence adversarial attack and defense technologies. Applied Sciences. 2019;9(5):909. doi: 10.3390/app9050909

[19] Shang J, Chen S, Wu J, Yin S. ARSpy: Breaking Location-based Multi-player Augmented Reality Application for User Location Tracking. IEEE Transactions on Mobile Computing. 2020. doi: 10.1109/TMC.2020.3007740

[20] Dantas Silva FS, Silva E, Neto EP, Lemos M, Venancio Neto AJ, Esposito F. A taxonomy of DDoS attack mitigation approaches featured by SDN technologies in IoT scenarios. Sensors. 2020;20(11):3078. doi: 10.3390/s20113078 32485943 PMC7309081

[21] Tariq N, Asim M, Al-Obeidat F, Zubair Farooqi M, Baker T, Hammoudeh M, et al. The security of big data in fog-enabled IoT applications including blockchain: A survey. Sensors. 2019;19(8):1788. doi: 10.3390/s19081788 31013993 PMC6515199

[22] Gadekallu TR, Manoj M, Kumar N, Hakak S, Bhattacharya S, et al. Blockchain-Based Attack Detection on Machine Learning Algorithms for IoT-Based e-Health Applications. IEEE Internet of Things Magazine. 2021;4(3):30–33. doi: 10.1109/IOTM.1021.2000160

[23] Fuentes-García M, Camacho J, Maciá-Fernández G. Present and Future of Network Security Monitoring. IEEE Access. 2021;9:112744–112760. doi: 10.1109/ACCESS.2021.3067106

[24] Gaur K, Kalla A, Grover J, Borhani M, Gurtov A, Liyanage M. A survey of virtual private LAN services (VPLS): Past, present and future. Computer Networks. 2021;196:108245. doi: 10.1016/j.comnet.2021.108245

[25] Kaur S, Gupta AK. Position based routing in mobile Ad-hoc networks: An overview. IJCST. 2012;3(4).

[26] Alzubi JA, Almomani O, Alzubi OA, Al-shugran M. Intelligent and dynamic neighbourhood entry lifetime for position-based routing protocol using fuzzy logic controller. International Journal of Computer Science and Information Security. 2016;14(1):118.

[27] Sultan K, Ali H, Zhang Z. Big data perspective and challenges in next generation networks. Future Internet. 2018;10(7):56. doi: 10.3390/fi10070056

[28] Jemmali M, Alquhayz H. Equity data distribution algorithms on identical routers. In: International Conference on Innovative Computing and Communications. Springer; 2020. p. 297–305.

[29] Katarahweire M, Bainomugisha E, Mughal KA. Data classification for secure mobile health data collection systems. Development Engineering. 2020;5:100054. doi: 10.1016/j.deveng.2020.100054

[30] Sarhan A, Lilien L. An Approach to identity management in clouds without trusted third parties. arXiv preprint arXiv:190400880. 2019. 10.48550/arXiv.1904.00880

[31] Burke Q, Mehmeti F, George R, Ostrowski K, Jaeger T, La Porta T, et al. Enforcing Multilevel Security Policies in Unstable Networks. IEEE Transactions on Network and Service Management. 2022. doi: 10.1109/TNSM.2022.3176820

[32] Ali F, Mathew S. An efficient multilevel security architecture for blockchain-based IoT networks using principles of cellular automata. PeerJ Computer Science. 2022;8:e989. doi: 10.7717/peerj-cs.989 35721416 PMC9202632

[33] Lin HY, Hsieh MY. A dynamic key management and secure data transfer based on m-tree structure with multi-level security framework for Internet of vehicles. Connection Science. 2022;34(1):1089–1118. doi: 10.1080/09540091.2022.2045254

[34] Achleitner S, Burke Q, McDaniel P, Jaeger T, La Porta T, Krishnamurthy S. MLSNet: A policy complying multilevel security framework for software defined networking. IEEE Transactions on Network and Service Management. 2020;18(1):729–744. doi: 10.1109/TNSM.2020.3045998

[35] Li X, Lyu MR, Liu J. A trust model based routing protocol for secure ad hoc networks. In: 2004 IEEE Aerospace Conference Proceedings (IEEE Cat. No. 04TH8720). vol. 2. IEEE; 2004. p. 1286–1295.

[36] Venkataraman R, Moeller S, Krishnamachari B, Rao TR. Trust–based backpressure routing in wireless sensor networks. International Journal of Sensor Networks. 2015;17(1):27–39. doi: 10.1504/IJSNET.2015.067591

[37] Tang J, Liu A, Zhao M, Wang T. An aggregate signature based trust routing for data gathering in sensor networks. Security and Communication Networks. 2018;2018. doi: 10.1155/2018/6328504

[38] Abd El-Moghith IA, Darwish SM. Towards designing a trusted routing scheme in wireless sensor networks: A new deep blockchain approach. IEEE Access. 2021;9:103822–103834. doi: 10.1109/ACCESS.2021.3098933

[39] Ramezan G, Leung C. A blockchain-based contractual routing protocol for the internet of things using smart contracts. Wireless Communications and Mobile Computing. 2018;2018. doi: 10.1155/2018/4029591

[40] Mayadunna H, De Silva SL, Wedage I, Pabasara S, Rupasinghe L, Liyanapathirana C, et al. Improving trusted routing by identifying malicious nodes in a MANET using reinforcement learning. In: 2017 Seventeenth International Conference on Advances in ICT for Emerging Regions (ICTer). IEEE; 2017. p. 1–8.

[41] Shukla A, Singh D, Sajwan M, Verma A, Kumar A. A source location privacy preservation scheme in WSN-assisted IoT network by randomized ring and confounding transmission. Wireless Networks. 2022;28(2):827–852. doi: 10.1007/s11276-021-02876-9

[42] Boneh D, Franklin M. Identity-based encryption from the Weil pairing. SIAM journal on computing. 2003;32(3):586–615. doi: 10.1137/S0097539701398521

[43] Ferraiolo DF, Kuhn DR, Chandramouli R. Role-Based Access Control. Artech House. Inc, Norwood, MA, USA. 2003.

[44] Goyal V, Pandey O, Sahai A, Waters B. Attribute-based encryption for fine-grained access control of encrypted data. In: Proceedings of the 13th ACM conference on Computer and communications security; 2006. p. 89–98.

[45] Sarhan AY, Carr S. A highly-secure self-protection data scheme in clouds using active data bundles and agent-based secure multi-party computation. In: 2017 IEEE 4th International Conference on Cyber Security and Cloud Computing (CSCloud). IEEE; 2017. p. 228–236.

[46] Melhim LKB, Jemmali M, Alharbi M. Network Monitoring Enhancement based on Mathematical Modeling. In: 2019 2nd International Conference on Computer Applications & Information Security (ICCAIS). IEEE; 2019. p. 1–4.

[47] Melhim LKB, Jemmali M, AsSadhan B, Alquhayz H. Network traffic reduction and representation. International Journal of Sensor Networks. 2020;33(4):239–249. doi: 10.1504/IJSNET.2020.109193

[48] Safaei M, Ismail AS, Chizari H, Driss M, Boulila W, Asadi S, et al. Standalone noise and anomaly detection in wireless sensor networks: a novel time-series and adaptive Bayesian-network-based approach. Software: Practice and Experience. 2020;50(4):428–446. doi: 10.1002/spe.2785

[49] A Ghaleb F, Saeed F, Al-Sarem M, Ali Saleh Al-rimy B, Boulila W, Eljialy A, et al. Misbehavior-aware on-demand collaborative intrusion detection system using distributed ensemble learning for VANET. Electronics. 2020;9(9):1411. doi: 10.3390/electronics9091411

[50] Ferchichi A, Boulila W, Farah IR. Reducing uncertainties in land cover change models using sensitivity analysis. Knowledge and Information Systems. 2018;55(3):719–740. doi: 10.1007/s10115-017-1102-9

[51] Boulila W, Sellami M, Driss M, Al-Sarem M, Safaei M, Ghaleb FA. RS-DCNN: A novel distributed convolutional-neural-networks based-approach for big remote-sensing image classification. Computers and Electronics in Agriculture. 2021;182:106014. doi: 10.1016/j.compag.2021.106014

[52] Ghandorh H, Boulila W, Masood S, Koubaa A, Ahmed F, Ahmad J. Semantic Segmentation and Edge Detection—Approach to Road Detection in Very High Resolution Satellite Images. Remote Sensing. 2022;14(3):613. doi: 10.3390/rs14030613

[53] Sarhan A, Jemmali M, Ben Hmida A. Two routers network architecture and scheduling algorithms under packet category classification constraint. In: The 5th International Conference on Future Networks & Distributed Systems; 2021. p. 119–127.

[54] Alharbi M, Jemmali M. Algorithms for investment project distribution on regions. Computational Intelligence and Neuroscience. 2020;2020. doi: 10.1155/2020/3607547 32802026 PMC7416242

[55] Melhim LKB, Jemmali M, Alharbi M. Intelligent real-time intervention system applied in smart city. In: 2018 21st Saudi Computer Society National Computer Conference (NCC). IEEE; 2018. p. 1–5.

[56] Jemmali M. Budgets balancing algorithms for the projects assignment. International Journal of Advanced Computer Science and Applications. 2019;10(11). doi: 10.14569/IJACSA.2019.0101177

[57] Alquhayz H, Jemmali M, Otoom MM. Dispatching-rule variants algorithms for used spaces of storage supports. Discrete Dynamics in Nature and Society. 2020;2020. doi: 10.1155/2020/1072485

[58] Haouari M, Gharbi A, Jemmali M. Bounding Strategies for Scheduling on Identical Parallel Machines. In: 2006 International Conference on Service Systems and Service Management. vol. 2. IEEE; 2006. p. 1162–1166.

[59] Haouari M, Hidri L, Jemmali M. Tighter Lower Bounds via Dual Feasible Functions. PMS 2008. 2008; p. 112.

[60] Hidri L, Jemmali M. Near-optimal solutions and tight lower bounds for the parallel machines scheduling problem with learning effect. RAIRO-Operations Research. 2020;54(2):507–527. doi: 10.1051/ro/2020009

